# Supplementary material for: Cross-disease modeling of peripheral blood identifies biomarkers of type 2 diabetes predictive of Alzheimer’s disease
Source: bioRxiv. 2024 Dec 12:2024.12.11.627991. Preprint. [Version 1] doi: 10.1101/2024.12.11.627991 (PMC11661382; doi:10.1101/2024.12.11.627991)
Supplement: Supplement 2 [file NIHPP2024.12.11.627991v1-supplement-2.pdf]

bioRxiv preprint doi: https://doi.org/10.1101/2024.12.11.627991; this version posted December 12, 2024. The copyright holder for this preprint (which was not certified by peer review) is the author/funder, who has granted bioRxiv a license to display the preprint in perpetuity. It is made available under aCC-BY 4.0 International license.

SUPPLEMENTARY INFORMATION

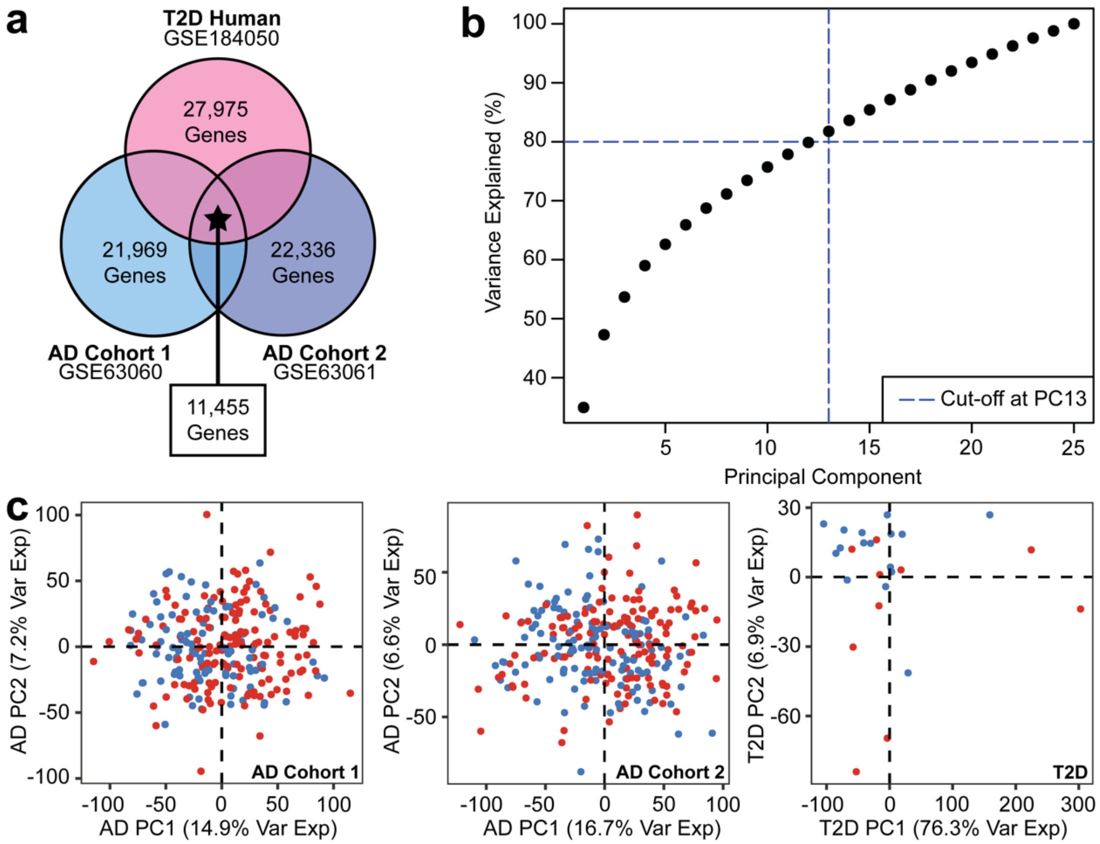

Supplementary Figure S1. Data processing of the AD and T2D data. **(a)** Gene overlaps across the three publicly available transcriptomics data. **(b)** Cumulative variance was explained for T2D PCs with a threshold of 80%. **(c)** PCA of the AD cohort 1, AD cohort 2, and T2D data.

bioRxiv preprint doi: https://doi.org/10.1101/2024.12.11.627991; this version posted December 12, 2024. The copyright holder for this preprint (which was not certified by peer review) is the author/funder, who has granted bioRxiv a license to display the preprint in perpetuity. It is made available under aCC-BY 4.0 International license.

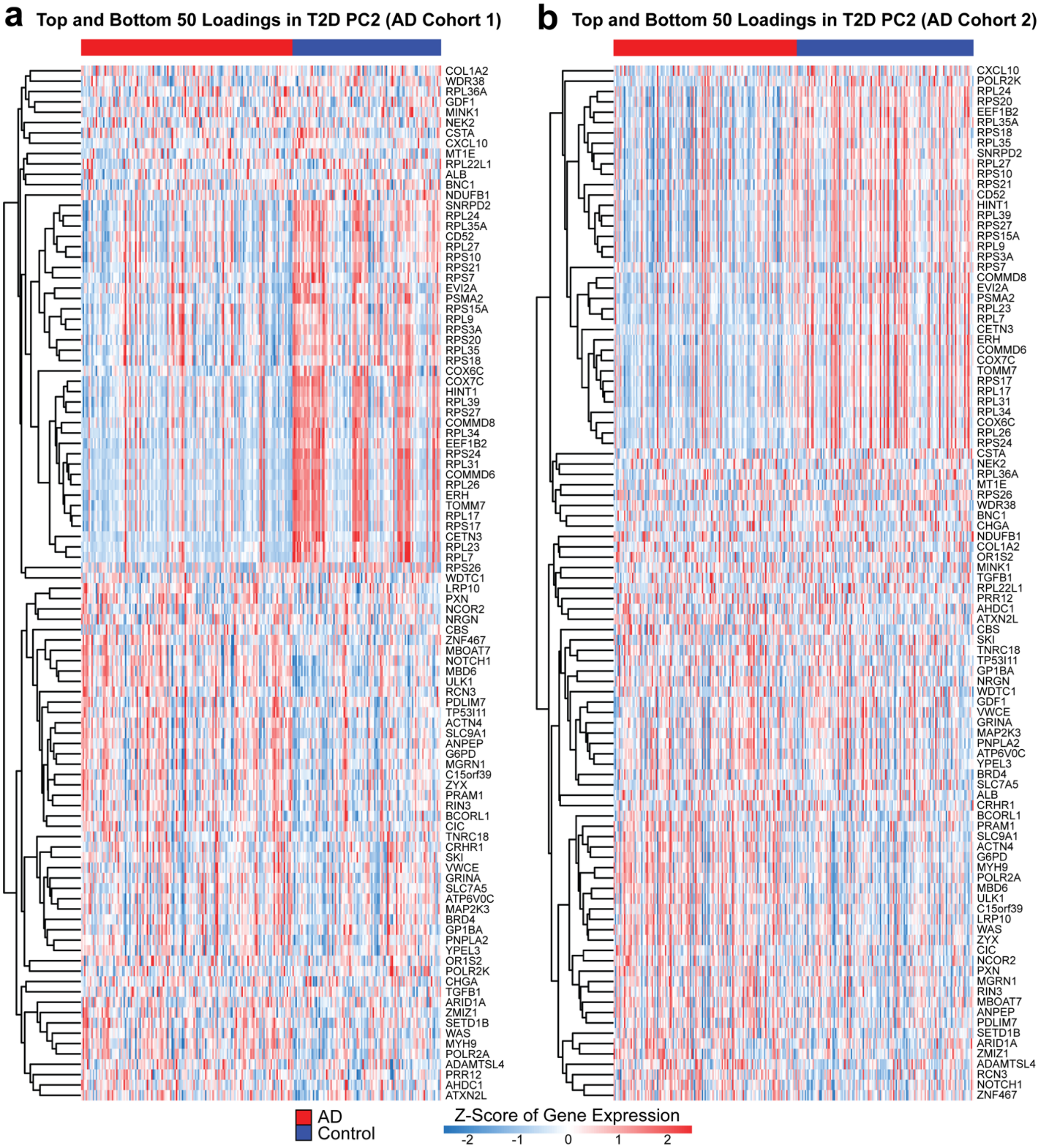

Supplementary Figure S2. **Identification of genes separating between healthy and AD groups.** Hierarchical clustering of the top and bottom 50 T2D PC2 loadings in T2D PC2 for **(a)** AD cohort 1 and **(b)** AD cohort 2.

bioRxiv preprint doi: https://doi.org/10.1101/2024.12.11.627991; this version posted December 12, 2024. The copyright holder for this preprint (which was not certified by peer review) is the author/funder, who has granted bioRxiv a license to display the preprint in perpetuity. It is made available under aCC-BY 4.0 International license.

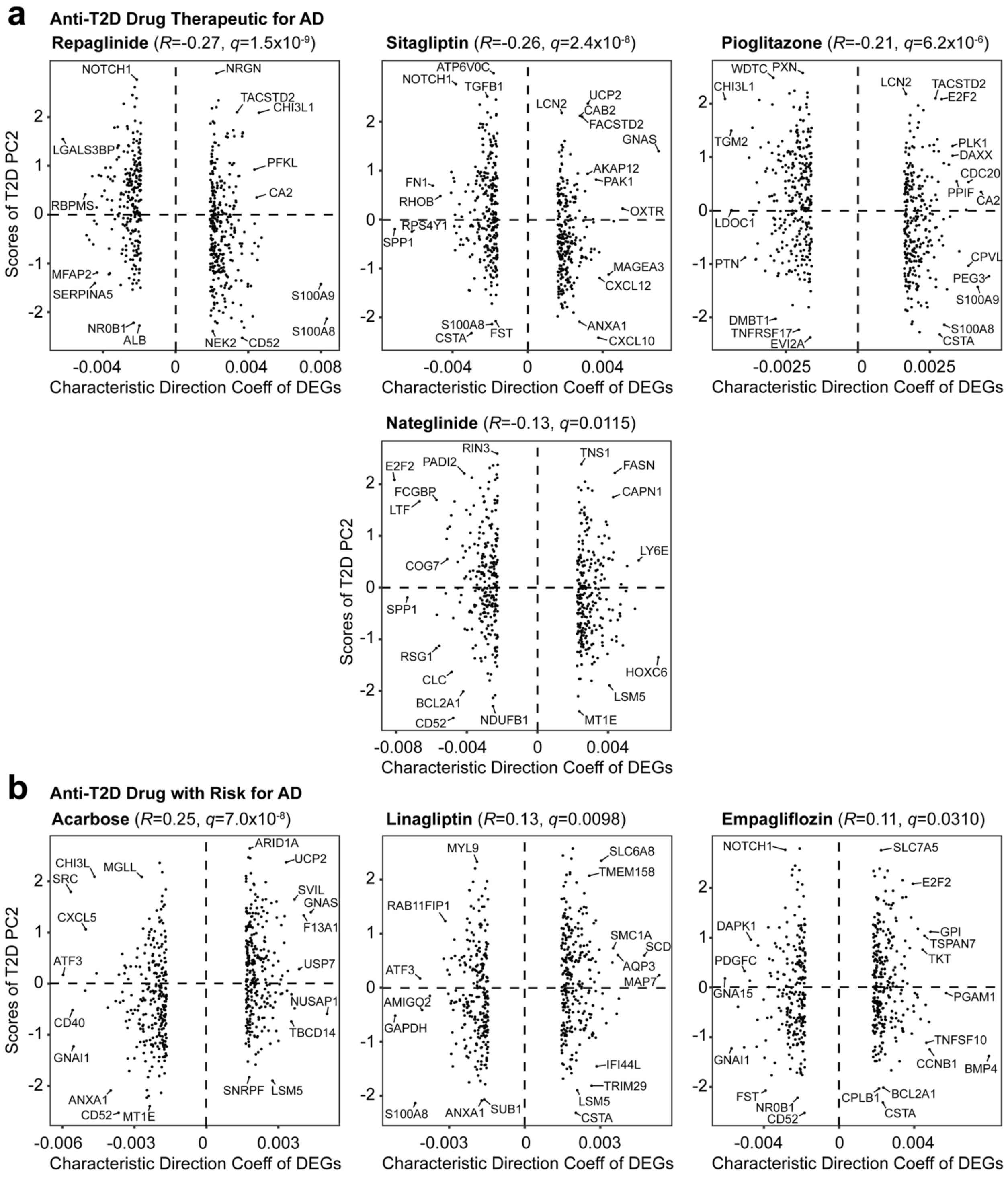

Supplementary Figure S3. Additional anti-T2D drugs approved by the FDA. (a) Anti-T2D therapeutics with potential reduction of AD pathology. **(b)** Anti-T2D therapeutics with increased risk for AD.

bioRxiv preprint doi: https://doi.org/10.1101/2024.12.11.627991; this version posted December 12, 2024. The copyright holder for this preprint (which was not certified by peer review) is the author/funder, who has granted bioRxiv a license to display the preprint in perpetuity. It is made available under aCC-BY 4.0 International license.

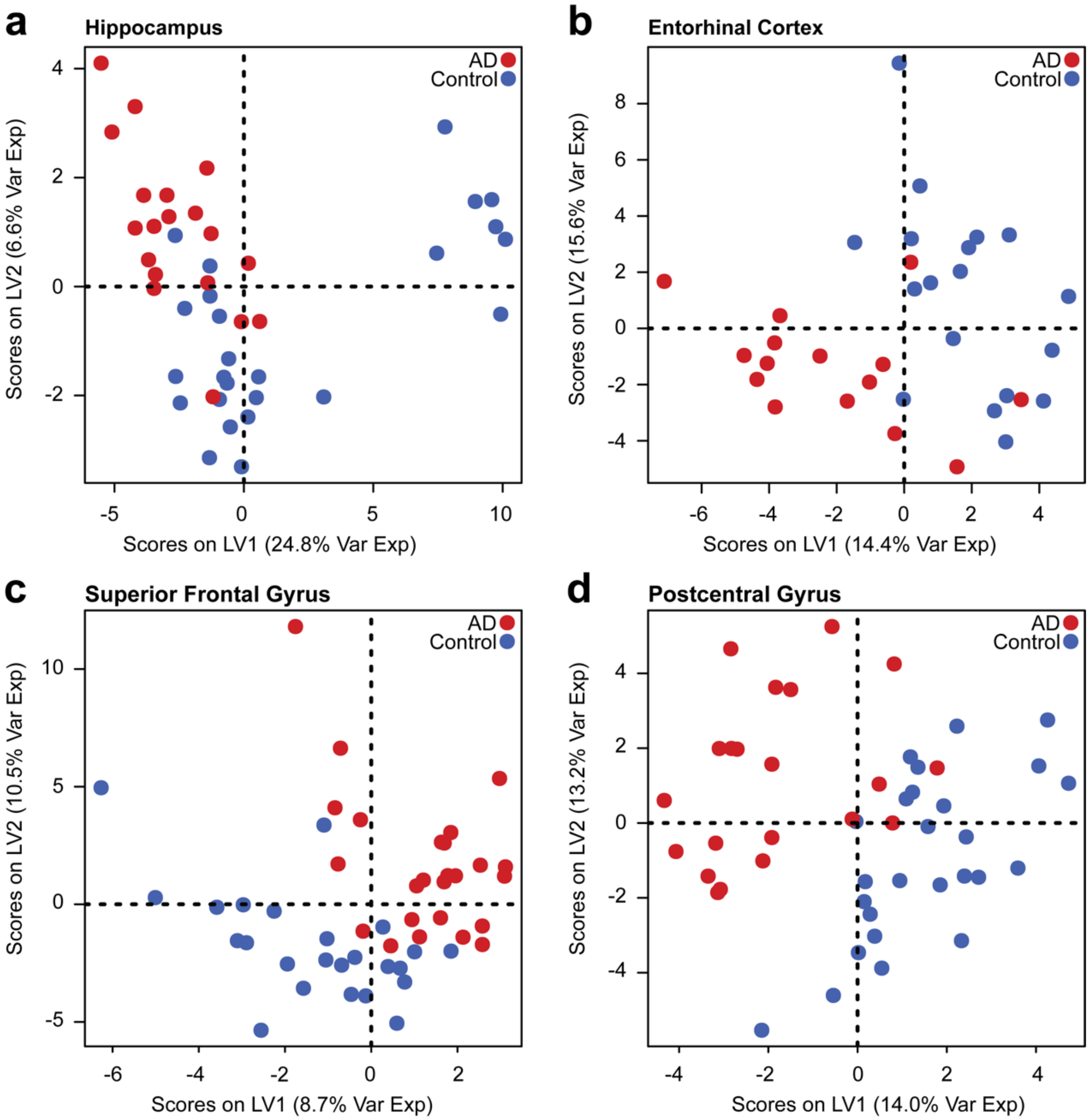

Supplementary Figure S4. PLS-DA models of the brain tissue gene expression filtered by the 88 represented in the T2D PC2 loading. Models constructed for **(a)** the hippocampus, **(b)** the entorhinal cortex, **(c)** the superior frontal gyrus, and **(d)** the postcentral gyrus.

bioRxiv preprint doi: https://doi.org/10.1101/2024.12.11.627991; this version posted December 12, 2024. The copyright holder for this preprint (which was not certified by peer review) is the author/funder, who has granted bioRxiv a license to display the preprint in perpetuity. It is made available under aCC-BY 4.0 International license.

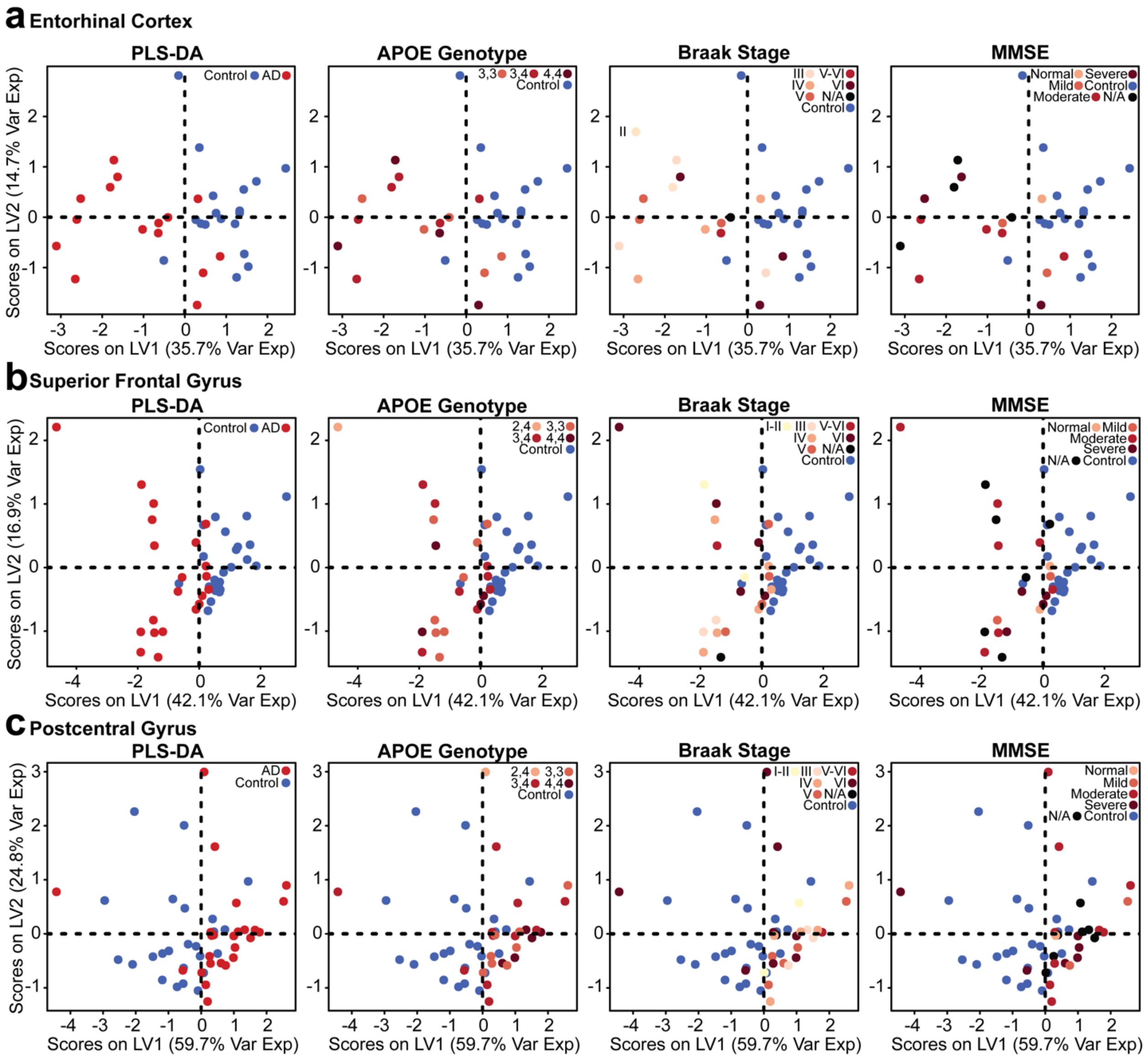

Supplementary Figure S5. Annotated PLS-DA models by genotype and clinical scores. Subjects are further labeled by their respective APOE genotype, Braak stage, and MMSE for brain tissue collected for the **(a)** entorhinal cortex, **(b)** superior frontal gyrus, and **(c)** postcentral gyrus.
